# Long-term sequelae are highly prevalent one year after hospitalization for severe COVID-19

**DOI:** 10.1038/s41598-021-01215-4

**Published:** 2021-11-22

**Authors:** Mattia Bellan, Alessio Baricich, Filippo Patrucco, Patrizia Zeppegno, Carla Gramaglia, Piero Emilio Balbo, Alessandro Carriero, Chiara Santa Amico, Gian Carlo Avanzi, Michela Barini, Marco Battaglia, Simone Bor, Vincenzo Cantaluppi, Giuseppe Cappellano, Federico Ceruti, Annalisa Chiocchetti, Elisa Clivati, Mara Giordano, Daria Cuneo, Eleonora Gambaro, Eleonora Gattoni, Alberto Loro, Marcello Manfredi, Umberto Morosini, Francesco Murano, Elena Paracchini, Giuseppe Patti, David James Pinato, Davide Raineri, Roberta Rolla, Pier Paolo Sainaghi, Stefano Tricca, Mario Pirisi

**Affiliations:** 1 Università del Piemonte Orientale UPO, Novara, Italy; 2 “AOU Maggiore Della Carità”, Novara, Italy; 3 Department of Surgery & Cancer, Imperial College London, Hammersmith Hospital, London, UK; 4 Department of Translational Medicine, Università del Piemonte Orientale UPO, Via Solaroli 17, 28100 Novara, NO Italy

**Keywords:** Microbiology, Virology

## Abstract

Many coronavirus disease 2019 (Covid-19) survivors show symptoms months after acute illness. The aim of this work is to describe the clinical evolution of Covid-19, one year after discharge. We performed a prospective cohort study on 238 patients previously hospitalized for Covid-19 pneumonia in 2020 who already underwent clinical follow-up 4 months post-Covid-19. 200 consented to participate to a 12-months clinical assessment, including: pulmonary function tests with diffusing lung capacity for carbon monoxide (DLCO); post-traumatic stress (PTS) symptoms evaluation by the Impact of Event Scale (IES); motor function evaluation (by Short Physical Performance Battery and 2 min walking test); chest Computed Tomography (CT). After 366 [363–369] days, 79 patients (39.5%) reported at least one symptom. A DLCO < 80% was observed in 96 patients (49.0%). Severe DLCO impairment (< 60%) was reported in 20 patients (10.2%), related to extent of CT scan abnormalities. Some degree of motor impairment was observed in 25.8% of subjects. 37/200 patients (18.5%) showed moderate-to-severe PTS symptoms. In the time elapsed from 4 to 12 months after hospital discharge, motor function improves, while respiratory function does not, being accompanied by evidence of lung structural damage. Symptoms remain highly prevalent one year after acute illness.

## Introduction

The acute phase of Coronavirus Disease 19 (Covid-19) has variable clinical manifestations and severity^1–3^. Weeks to months after acute illness, Covid-19 survivors may show persistent residual symptoms and organ impairment. Recently, the term “long Covid” has been proposed to identify this condition^4^. Long-lasting consequences of infection by severe acute respiratory syndrome coronavirus 2 (SARS-CoV-2) mirror evidence of organ damage documented for earlier coronaviruses epidemics: the severe acute respiratory syndrome coronavirus (SARS-CoV) and the Middle East respiratory syndrome coronavirus (MERS-CoV), belonging to the same *Betacoronavirus* genus as the SARS-CoV-2^5,6^.

Evolving evidence suggests the most common persistent physical symptoms following acute Covid-19 to include fatigue, dyspnea, chest pain, and cough^7,8^. Less commonly, patients may report long-term osteo-articular complaints and neurosensorial alterations^9,10^. Functional respiratory impairment has been observed months after severe SARS-CoV-2 infection as a consequence of diffuse alveolar and capillary damage, hyaline membrane formation, alveolar septal fibrous proliferation, and pulmonary consolidation^11^.

Reduction in the diffusion capacity of the lung for carbon monoxide (DLCO) is the most common functional consequence of severe SARS-CoV-2, occurring typically during the acute phase^12^ and persisting up to six months post-recovery in up to 50% of Covid-19 survivors^7,13^. Alongside respiratory dysfunction, motor impairment is often reported post-Covid-19. When assessed with a 6 min walking test (6MWT), a vast proportion of survivors to Covid-19 walked a distance lower than expected for their age-adjusted predicted values^14^. Similar results have been reported with other tools, such as the Short Physical Performance Battery (SPPB) or the 2-min walk test (2MWT)^15^. Finally, the impact of Covid-19 on mental health should not be neglected: according to different studies, anxiety and/or depression may be reported in up to 30% of patients months after recovery^9,16^ and post-traumatic symptoms are also quite common^7^.

Whilst a number of reports have provided an initial account of the post-Covid-19 sequelae, how these clinical manifestations evolve over time is uncertain. We previously described the early psychological and functional sequelae of Covid-19 infection 4 months after hospital discharge in a prospective cohort of 238 patients^13^. In this prospective observational study, we describe the long-term functional impairment of respiratory, motor, psychological and cognitive function 12 months after SARS-CoV-2 infection and compare these to the symptomatic burden at 4 months follow-up, in an attempt to clarify reversibility of symptomatology.

## Results

### General features of the study population

The main characteristics of participants are listed in Table 1. The median follow-up time was 366 days [363–369]. In the time interval between month 4 and 12 months, only 1 patient re-tested positive for SARS-Cov-2 although he remained completely asymptomatic. At the 12-month time point, 79 patients (39.5%) reported at least one symptom. While the proportion of patients complaining dyspnea (p = 0.40), dysgeusia (p = 0.63), anosmia (p = 0.05), fatigue (p = 0.66), sore throat (p = 1.0) and chest pain (p = 1.0) did not change between the 4 and 12 months visit, interestingly, more patients complained cough (p < 0.0001) and arthralgia/ myalgia (p < 0.0001) at the 1 year follow-up visit.

**Table 1:** General features of study population.

**Demographic characteristics**			
Age, years		62 [51–71]	
Gender, females/males		78 (39.0)/122 (61.0)	
BMI, kg/m^2^		27.5 [24.6–31.6]	
Number of comorbidities		2 [1–3]	
CIRS		2 [1–3]	
Smoking attitude, no/former/active		111 (55.5)/66 (33.0)/23 (11.5)	
Pack-years		15 [7–30]	
**Severity of acute illness**			
Length of hospital in-stay, days		9 [5–16]	
Disease severity class			
Class 3		46 (23.0)	
Class 4		10 (5.0)	
Class 5		78 (39.0)	
Class 6		45 (22.5)	
Class 7		21 (10.5)	
**Maximal oxygen supplementation**			
No supplementation		59 (29.5)	
Nasal cannulae or venturi mask		82 (41.0)	
Non-invasive ventilation		41 (20.5)	
Mechanical ventilation		18 (9.0)	
ICU admission		23 (11.5)	
Length of ICU, days		10 [6–21]	
**Comorbidities**			
Arterial hypertension		82 (41.0)	
Diabetes		31 (15.5)	
Dyslipidemia		18 (9.0)	
COPD		12 (6.0)	
Obesity		22 (11.0)	
IBD		4 (2.0)	
Chronic liver disease		7 (3.6)	
Autoimmune disease		3 (1.5)	
Hematological disease		13 (6.5)	
Coronary artery disease		18 (9.0)	
Atrial fibrillation		13 (6.5)	
Other structural heart disease		3 (1.5)	
Other arrhythmogenic heart disease		6 (3.0)	
Endocrinological disease		22 (11.0)	
CKD		12 (6.0)	
Stroke/TIA		5 (2.5)	
VTE		4 (2.0)	
Anxiety and depression		8 (4.0)	
Active malignancy		18 (9.0)	

**Symptoms**			
	During the acute phase	At 4 months follow-up	At 12 months follow-up
Fever	184 (92.0)	0 (0.0)	0 (0.0)
Cough	112 (56.0)	4 (2.0)	22 (11.2)
Dyspnea	129 (54.2)	13 (5.5)	16 (8.1)
mMRC	N/A	N/A	
Class 0			121 (61.7)
Class 1			43 (21.9)
Class 2			16 (8.2)
Class 3			14 (7.1)
Class 4			2 (1.0)
Dysgeusia	62 (31.0)	10 (5.0)	13 (6.6)
Anosmia	55 (27.5)	10 (5.0)	19 (9.7)
Arthralgia/myalgia	46 (19.3)	13 (6.5)	43 (21.9)
Chest pain	2 (1.0)	0 (0.0)	0 (0.0)
Sore throat	1 (0.4)	0 (0.0)	0 (0.0)
Alopecia	N/A	N/A	71 (36.2)
Fatigue	72 (36.0)	34 (17.0)	30 (15.4)

The table shows the main demographic features, severity of acute illness parameters, frequencies and percentage of patients reporting specific comorbidities and complaining any specific symptom of Covid-19 in the study population at baseline and at the follow-up visits. Categorical variables are shown as frequencies (%), while continuous variables are shown as medians and interquartile range [IQR]. *BMI* Body Mass Index, *CIRS* cumulative illness rating scale, *ICU* Intensive Care Unit, *COPD* Chronic obstructive pulmonary disease, *IBD* Inflammatory bowel diseases, *CKD* Chronic kidney disease, *TIA* Transient ischemic attack, *VTE* Venous thromboembolism, *mMRC* modified Medical Research Council questionnaire, *N/A* not available.

### Radiological assessment

A total of 190 patients consented to undergo a chest CT scan, while 10 refused; the median CT severity score was 2 [0–5]; a baseline CT scan performed during the acute phase was available for 111 patients. The score was significantly greater at baseline (6 [3–9]; p < 0.0001).

We also stratified patients according to different categories of radiological severity:

- None to mild involvement: score ≤ 5: N = 146 (76.8%);
- Moderate involvement: score > 5 and ≤ 10: N = 26 (13.7%);
- Severe involvement > 10: N  = 18 (9.5%).

### Pulmonary function tests

Four patients were not able to complete PFTs. As reported in eTable 1, the median FEV1 (101% [92–112]) and FVC (98% [90–109]) were normal and comparable to the 4-months follow-up. Similarly, the median DLCO did not change significantly over time, being at the lower limit of normality at 12-months (80% [69–91]). DLCO was < 80% of predicted value in 96 patients (49.0%); a more severe impairment (DLCO < 60% of predicted) was observed in 20 (10.2%) subjects. The prevalence of DLCO reduction below the considered thresholds did not change appreciably over time (also see eTable 1). Focusing on patients with altered DLCO at 12 months, 21.7% (N = 20) showed a significant improvement (DLCO increase > 5%) with respect to 4-months follow-up visit, while 29.3% (N = 27) showed a significant worsening (DLCO reduction > 5%) (also see eTable 2 and eFig. 2 for more details).

In eTables 3 and 4 we report the results of the univariate analysis of factors associated with impaired DLCO. As shown in Table 2, at logistic regression, female gender was strongly associated with a DLCO < 80% at one-year follow-up, independently of other factors, whereas arterial hypertension, chronic kidney disease and radiological involvement were associated with having a DLCO < 60% at the 12-month time-point.

**Table 2:** Logistic regression for DLCO impairment.

	OR	p
**DLCO < 80%**		
Gender	4.48 [2.05–9.81]	0.0002
Age	1.01 [0.98–1.04]	0.52
CAD	2.97 [0.74–11.93]	0.13
CIRS	1.18 [0.80–1.75]	0.41
ICU	0.93 [0.17–5.15]	0.93
Modality of oxygen delivery	1.17 [0.53–2.60]	0.70
Length of hospital in stay	1.04 [0.99–1.09]	0.07
Smoke	1.16 [0.77–1.75]	0.47
Severity of acute illness	1.09 [0.66–1.80]	0.75
CT severity score	2.07[0.98–4.37]	0.06
IES	1.01 [0.99–1.04]	0.20
CKD	5.40 [0.88–33.19]	0.07
Persistent dyspnea	1.88 [0.45–7.80]	0.38
**DLCO < 60%**		
T2DM	1.85 [0.49–6.87]	0.36
Arterial hypertension	3.91 [1.00–15.28]	0.05
CIRS	1.88 [0.85–4.19]	0.11
CKD	10.05 [1.93–52.41]	0.006
Length of hospital in stay	1.01 [0.96–1.06]	0.58
CT severity score	3.03 [1.24–7.37]	0.01
COPD	4.32 [0.58–32.38]	0.15
Smoke	1.05 [0.54–2.07]	0.86
Class severity	0.94 [0.53–1.67]	0.84

Multivariable logistic regression. Odds Ratio (OR) with CI95% and P-values (P) are reported. In the upper part of the table we reported the model for the prediction of a reduction of DLCO under the threshold of 80%; in the lower part we reported a model predicting a more severe functional impairment (DLCO < 60%). *CAD* coronary artery disease, *CIRS* cumulative illness rating scale, *ICU* Intensive Care Unit, *CT* computed tomography, *IES* Impact of event scale, *CPDI* CoViD-19 Peritraumatic Distress Index, *T2DM* type 2 diabetes mellitus, *COPD* chronic obstructive pulmonary disease, *CKD* Chronic kidney disease.

Patients complaining of dyspnea at rest or for mild efforts (mMRC ≥ 3; N. = 16 (8.1%) patients) were significantly older (70.5 [55.5–77.5] vs. 61.0 [50.5–70.0]; p = 0.04), with a higher BMI (31.2 [26.9–33.2] vs. 27.0 [24.6–31.3]; p = 0.04) and signs of persistent mental health distress (IES: 17 [7.5–41.5] vs. 6 [7–19], p = 0.01; CPDI: 17 [7.5–35.5] vs. 9 [3–17], p = 0.04); moreover, they showed a lower DLCO (67 [59–78] vs. 81 [70–91]; p = 0.01), although the CT severity score did not differ between groups (p = 0.49). Prevalence of dyspnea was higher in females (p = 0.01).

### Physical performance evaluation

2 patients refused to perform physical assessment. The SPPB test was altered in 37/198 (18.7%) subjects. All other patients (N = 161) underwent a 2MWT; 14 patients (7.1%) walked a distance lower than expected for age and gender. Thus, 51 patients (25.8%) had some degree of functional impairment. None of them reported a new diagnosis of inflammatory arthritis or myopathy. This proportion is significantly lower than that reported in the same group of patients 4 months after hospital discharge (N = 103, 52.8%; − 27% CI95 (− 33; − 20%); p < 0.001).

In eTable 5 we report the results of univariate analysis. At logistic regression (Table 3), DLCO and COPD were independently associated with motor impairment.

**Table 3:** Logistic regression for motor function impairment.

	OR	p
**Motor function impairment**		
Gender	1.93 [0.70–5.30]	0.20
Age	1.02 [0.98–1.06]	0.30
Obesity	0.38 [0.06–2.32]	0.30
BMI	1.05 [0.97–1.14]	0.29
CIRS	1.40 [0.77–2.51]	0.27
Smoke	0.72 [0.42–1.23]	0.23
OSAS	2.06 [0.13–233.70]	0.61
IES	1.02 [0.99–1.05]	0.15
T2DM	2.06 [0.66–6.39]	0.21
Arterial hypertension	2.00 [0.78–5.15]	0.15
CKD	0.76 [0.13–4.56]	0.76
Atrial fibrillation	1.60 [0.34–7.53]	0.55
CT severity score	0.70 [0.35–1.40]	0.31
COPD	6.51 [1.14–37.23]	0.04
DLCO	0.96 [0.92–0.99]	0.01
Arthralgia/myalgia	2.37 [0.80–6.95]	0.12

Multivariable logistic regression. Odds Ratio (OR) with CI95% and P-values (P) are reported. *BMI* body mass index, *CIRS* cumulative illness rating scale, *OSAS* obstructive sleep apnea syndrome, *IES* Impact of event scale, *T2DM* type 2 diabetes mellitus, *CKD* Chronic kidney disease, *CT* computed tomography, *COPD* chronic obstructive pulmonary disease, *DLCO* diffusion capacity of the lung for carbon monoxide.

Thirty patients reported fatigue, although the number raised to 66 (33.3%), when this symptom was assessed by the CFS. A CFS consistent with fatigue was associated to: female gender (p = 0.0008); a higher BMI (29.5 [25.0–32.9] vs. 26.7 [24.4–32.9]; p = 0.04) and a higher level of mental health distress (IES: 12 [4–27] vs. 4 [1–15], p = 0.001); moreover, they showed a lower DLCO (75 [65–82] vs. 82 [71–93]; p = 0.003). The CT severity score (p = 0.15) and age (p = 0.31) did not differ between groups.

### Mental health symptoms tests

Answers to the IES questionnaire revealed the presence of mild, moderate and severe symptoms in 48 (24.0%), 24 (12.0%) and 13 (6.5%) patients, respectively. These rates were in line with those reported in the same cohort evaluated at 4 months (25.1%, 11.6% and 5.0% respectively; the presence of moderate to severe PTS symptoms was, indeed, 18.5% at 4 months and 16.6% at 12 months, p = 0.69). None of the factors included in the logistic regression analysis was independently associated with the presence of moderate to severe PTS symptoms (eTable 6 and Table 4).

**Table 4:** Logistic regression for post-traumatic stress symptoms.

	OR	p
**IES > 25**		
Gender	1.95 [0.87–4.37]	0.10
BMI	1.04 [0.98–1.10]	0.16
Atrial fibrillation	2.03 [0.59–7.07]	0.26
Length of hospital in stay	1.01 [0.98–1.04]	0.40
DLCO	0.98 [0.96–1.01]	0.36
CT severity score	1.22 [0.66–2.25]	0.51

Multivariable logistic regression. Odds Ratio (OR) with CI95% and P-values (P) are reported. In the table we reported the model for the prediction of moderate to severe PTS symptoms according to the IES. *IES* Impact of event scale, *CPDI* CoViD-19 Peritraumatic Distress Index, *BMI* body mass index, *DLCO* diffusion capacity of the lung for carbon monoxide.

## Discussion

Since the first outbreak of SARS-Cov-2 in Wuhan great emphasis has been devoted to the understanding of determinants of acute morbidity and mortality from Covid-19. More recent studies have highlighted the long-term consequences from SARS-Cov-2 infection in patients who survive severe disease, however data on long-term impact and reversibility of Covid-19-related sequelae are lacking.

This is one of the first studies assessing the persistence of symptoms from severe Covid-19 one year after hospital discharge; according to our data, symptoms may persist in approximately 40% of patients. In a very recent report by Lombardo et al., the proportion of patients still complaining symptoms after 12 months is even higher (81%)^17^. In a large Chinese prospective cohort, Huang et al. reported that 76% of patients complained of at least one residual symptom 6 months after acute illness^9^, although lower rates (around 40–50%) have been reported by other authors^7,18,19^. These observations are clues that Covid-19 may cause persistent organ damage. However, symptoms are, by definition, subjective and might be influenced by psychological factors. This might also explain why some symptoms, such as cough and arthromyalgia were even more common at 12 than at 4 months. It does not probably reflect a progressive worsening of long Covid, rather suggesting a deeper insight of the general population about this issue. Indeed, the second time point visit of our study was carried out while the media were already informing the population about the longstanding consequences of SARS-CoV-2 infection, which were less known when we carried out the first time point visit. Thus, these differences are probably due to the fact that patients paid more attention to their symptoms.

Covid-19 pandemic has had a strong detrimental impact on the mental health of general population; according to a large international cohort of almost 10,000 subjects, the pandemic was experienced as at least moderately stressful for most people while 11% reported the highest levels of stress. Moreover, symptoms of depression were common; lack of social support, low education level, and precarious financial status were all predictors of worse psychological outcomes^20^. Restrictions to free movement and social distancing makes it difficult to estimate the background psychological impact of Covid-19 at population level. According to our data, both fatigue and dyspnea are associated to the IES score, suggesting that these physical symptoms and the mental health trauma derived from Covid-19 may have a role in the persistence of these symptoms. This is important, if we consider that in our cohort, the prevalence of subjects experiencing moderate to severe PTS symptoms did not significantly decrease over time, remaining stable from 4 to 12 months after discharge. Our data are in line with those of other authors suggesting a prevalence of PTS symptoms around 20% in patients recovered from Covid-19^21,22^ and reflects, at least in part, the high level of stress in the population. Indeed, accordingly to a recent meta-analysis, the prevalence of PTS disorder in the general population during the pandemic is around 15%^23^.

In addition, impaired alveolar gas transfer assessed by DLCO observed in the first months following SARS-CoV-2 infection, did not show any appreciable improvement over time. This objective functional alteration is related to an anatomical damage^24^. Indeed, the CT severity score is a major determinant particularly for severe DLCO reduction, as reported previously^25^. This suggests that a structural damage subtends the development of respiratory impairment and is involved in the pathogenesis of dyspnea. However, as previously mentioned, the observation that the proportion of patients complaining dyspnea increased significantly over time, despite a stable DLCO, makes evident that this symptom has a relevant psychological background, as further confirmed by its association with IES. Therefore, we can summarize that beside patients with respiratory impairment, others may complain dyspnea mainly on a psychological basis.

Moreover, consistently with our previous findings^13^, DLCO reduction is strongly associated to motor impairment, suggesting a central role for respiratory system in driving physical functional limitations. Interestingly, althogh DLCO did not improve over time, we observed a significant reduction of patients with motor limitations from 4 to 12 months.

The improvement in motor function might reflect a beneficial effect of physical activity on around half of the patients; however, this aspect was not directly investigated and future research should help clarify whether exercise might improve long-term outcome from Covid-19.

Finally, our paper contributes to profiling the patients at higher risk for long-lasting sequelae. Different authors already reported that females are at higher risk for persistent symptoms and psychological involvement^8,9,26^, leading them to postulate a potential role for hormonal factors in the pathogenesis of this condition^27^. Our data are in line with the current literature, showing that female gender was associated to DLCO reduction, persistent dyspnea and fatigue. However, males have a poorer prognosis during acute illness^28^; hence, females may survive more frequently to severe Covid-19, possibly justifying the persistence of clinical sequelae. This is a possible explanation, although probably not completely satisfactory; indeed, when included in a multivariate model, parameters assessing acute disease severity were not related to DLCO reduction. Conversely, some specific comorbidities are associated to persistent functional impairment, such as COPD, CKD and arterial hypertension, which is a well-described negative prognostic factor during the acute phase^29^.

The main limitation of our study is the unavailability of detailed clinical data (particularly PFTs) predating SARS-CoV-2 infection, preventing us to discriminate the damage attributable to Covid-19 from that pre-existing this disease. Although we demonstrated that DLCO reduction was associated to the extent of radiological damage, the absence of CT scans before the infection did not allow to estimate whether the structural damage is consistent with the evolution of the acute illness or the result of respiratory comorbidities. A second limitation is the relatively small sample size; and a drop-out rate of 16% compared to the 4 month follow up timepoint. This was expected, also considering that the current follow-up visit coincided with an outbreak of the pandemic in March–April 2021. Despite the acknowledged limitations, the percentage of patients attending the one-year follow-up visit was > 80%, being representative of the original cohort.

In conclusion, symptoms of Covid-19 may persist up to 12 months after hospital discharge as a consequence of both mental stress and organ damage. Altered respiratory function secondary to radiologically proven structural lung damage and mental health sequelae persist over time in a significant proportion of patients. Conversely, the recovery of motor function continues during the follow-up from 4 to 12 months. Female gender, arterial hypertension, chronic kidney disease and COPD may represent risk factors for persistent Covid-19 sequelae.

## Methods

### Study population

**Figure 1:**
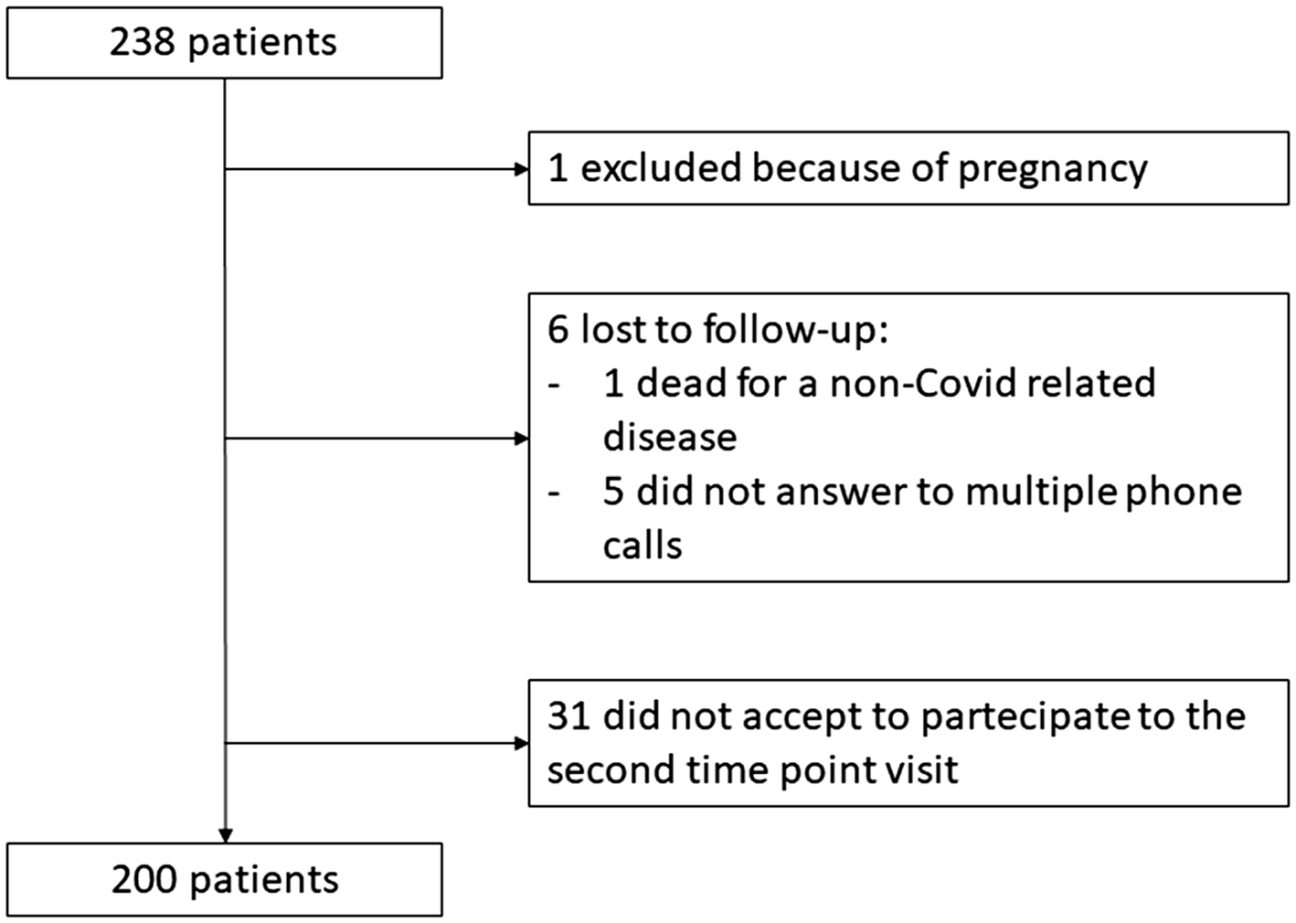
Flow-chart of the study. The figure shows how the study population was selected.

Briefly, as described in the previously published paper^13^, we contacted all the 767 patients (or their caregivers) aged 18 years or older who were discharged between March 1 and June 29, 2020, from the Azienda Ospedaliero–Universitaria Maggiore della Carità university hospital in Novara, Italy, Northern Italy, where they had been admitted for a confirmed diagnosis of Covid-19. Out of them 238 consented to participate to a follow-up visit performed 3 to 4 months after discharge; we contacted them again to schedule a further visit after 12 months. Patient disposition is reported in Fig. 1.

One patient was excluded because she was pregnant, while 6 more patients were lost to the follow-up. Thirty-one declined participation to the second time-point of the study (see Fig. 1 for further details) mainly because of impossibility to attend the visit (health or work issues) and/or fears of new in-hospital exposure to SARS-CoV-2. The eligible population included a total 200 patients, 84% of the originally recruited cohort, who were hospitalized between 1st March 2020 and 15th May 2020.

All patients were contacted telephonically and offered a clinical evaluation 12 months after hospital discharge: the visits were scheduled between 15th March and 4th May 2021.

All participants signed an informed consent and underwent a Complete clinical assessment including physical examination to investigate:

- Severity of acute illness, classified using an eight-category scale, as previously published^30^;
- Symptoms at SARS-CoV-2 diagnosis and at the 4-months evaluation;
- Co-morbidities, home medications and cumulative illness rating scale (CIRS)^31^;
- Patients’ symptoms at follow-up visit. Dyspnea was assessed using the modified Medical Research Council dyspnea scale (mMRC)^32^, while fatigue was assessed with the Chalder fatigue scale (CFS), categorizing patients based on a CFS score ≥ 4^33^.

The clinical evaluation was performed by hospital specialists involved in the management of Covid-19 patients and data recorded in an electronic case report form.

The study protocol was approved by the local ethical committee: Comitato Etico Interaziendale Novara (IRB code CE 117/20) and conducted in strict accordance with the principles of the Declaration of Helsinki. All the subjects who participate to the study signed a written informed consent.

The present report follows STROBE guidelines for cohort studies.

### Pulmonary function tests

All patients underwent standard pulmonary function testing (PFT) (with a Quark PFT with X9 pneumotach, COSMED srl, Roma, Italy) for forced expiratory volume in one second (FEV1), vital capacity (VC), forced vital capacity (FVC), DLCO and total lung capacity (TLC). DLCO and TLC were determined by the single-breath carbon monoxide technique. The examination was performed on the same day of the visit.

### Physical performance tests

On the same day, we also assessed the patient's physical performance; patients underwent a Short Physical Performance Battery (SPPB) as previously described: the score is considered as pathological when 10 or lower^34,35^.

For those patients with SPPB score > 10 we also performed a 2-min walk test (2MWT), which was compared with reference data values for an age-matched population, aiming to disclose more subtle functional alterations^36–38^.

### Mental health symptoms tests

We assessed the prevalence of post-traumatic stress (PTS) symptoms by administering the Impact of Event Scale (IES)^39^. A total score between 0 and 8 is interpreted as subclinical PTS; 9- 25 as mild PTS; 26–43 as moderate PTS and > 44 as severe PTS. Internal consistency coefficients for intrusion is 0.84, for avoidance is 0.71^40^.

### Radiological assessment

Chest computed tomography (CT) scans were performed during single full inspiratory breath hold in supine position on a 256-slice CT (PHILIPS Brilliance ICT). All images were reconstructed with a slice thickness of 1 mm.

The images in DICOM (Digital Imaging and COmmunications in Medicine) extension files were transferred to the Picture Archiving and Communication System (PACS) and then analyzed into a workstation equipped with two 35 × 43 cm monitors (produced by Eizo, with 2048 × 1536 matrix).

All CT scans have been evaluated by a radiologist with more than 10 years of experience in chest CT scan, blinded to clinical data. Both lung (width, 1600 HU; level, − 550 HU) and mediastinal (width, 400 HU; level, 40 HU) window settings were evaluated.

According to Han et al.^41^ and to Fleischner Society glossary^42^, the evidence of fibrotic-like changes was defined as the presence of honeycombing, bronchiectasis, pleural traction and parenchymal bands. Each of the five pulmonary lobes was visually scored from 0 to 5 as (0) no involvement, (1) less than 5% involvement, (2) 5–25% involvement, (3) 26–50% involvement, (4) 51–75% involvement, and (5) 76–100% involvement. The scores were summed to provide a total CT severity score ranging from 0 (no involvement) to 25 (maximum involvement; eFig. 1).

### Statistical analysis

Data were analysed using the statistical software Stata 15.1 (StataCorp, College Station, USA). Normality was assessed by Shapiro–Wilk test. The measures of centrality and dispersion for continuous variables were medians and [25th–75th percentile]; comparisons between groups for these variables were performed by the Mann–Whitney test. The median values of respiratory functional parameter were compared by Wilcoxon’s test for paired samples; the prevalence of DLCO reduction below the thresholds considered and the prevalence of physical impairment between 4 and 12 months were compared by McNemar’s test. Categorical variables, whenever dichotomous or nominal, were reported as frequencies and percentages, and analyzed through the Pearson’s chi-square or Fisher’s exact test, as appropriate. The primary endpoint was the proportion of patients with a DLCO < 80% of predicted value. The original cohort was sufficiently powered to detect as statistically significant a 0.12 increase in the proportion of patients with DLCO < 80% of predicted among Covid-19 survivors compared to that observed in a reference population (0.30 vs. 0.18, respectively), with an alpha level = 0.005. Secondary endpoints were: proportion of subjects showing severe DLCO (< 60%) impairment, potentially associated to a higher risk of pulmonary fibrosis; prevalence of motor impairment (SBBP score < 11 or SBBP score ≥ 11 and altered 2MWT); prevalence of moderate-to-severe PTS symptoms (IES > 26 and CDPI > 28); factors associated to the previous endpoints.

To disclose associations, we run a univariate analysis, including: the comorbidities with a biological rationale and CIRS, age, gender, smoking attitude, intensive care unit (ICU) admission during hospital in-stay, length of hospital in-stay, modality of oxygen delivery during hospital in-stay, class severity of acute illness, CT severity score. All variables associated with the outcome of interest at a p-value threshold below 0.20 were included in logistic regression models.

## Supplementary Information

Supplementary Information.

## Data Availability

All the data are available upon request to the corresponding author.

## References

[1] Chen N (2020). Epidemiological and clinical characteristics of 99 cases of 2019 novel coronavirus pneumonia in Wuhan, China: A descriptive study. Lancet.

[2] Bellan M (2020). Fatality rate and predictors of mortality in an Italian cohort of hospitalized COVID-19 patients. Sci. Rep..

[3] Polverino F (2020). Comorbidities, cardiovascular therapies, and COVID-19 mortality: A nationwide, Italian observational study (ItaliCO). Front. Cardiovasc. Med..

[4] Norton A (2021). Long COVID: Tackling a multifaceted condition requires a multidisciplinary approach. Lancet Infect Dis..

[5] Mann R (2020). Clinical characteristics, diagnosis, and treatment of major coronavirus outbreaks. Front. Med..

[6] O'Sullivan O (2021). Long-term sequelae following previous coronavirus epidemics. Clin. Med..

[7] Venturelli S (2021). Surviving COVID-19 in Bergamo province: A post-acute outpatient re-evaluation. Epidemiol Infect..

[8] Sykes DL (2021). Post-COVID-19 symptom burden: What is long-COVID and how should we manage it?. Lung.

[9] Huang C (2021). 6-month consequences of COVID-19 in patients discharged from hospital: A cohort study. Lancet.

[10] Lucidi D (2021). Patient-reported olfactory recovery after SARS-CoV-2 infection: A 6-month follow-up study. Int. Forum Allergy Rhinol..

[11] Xu Z (2020). Pathological findings of COVID-19 associated with acute respiratory distress syndrome. Lancet Respir. Med..

[12] Mo X (2020). Abnormal pulmonary function in COVID-19 patients at time of hospital discharge. Eur. Respir. J..

[13] Bellan M (2021). Respiratory and psychophysical sequelae among patients with COVID-19 four months after hospital discharge. JAMA Netw. Open..

[14] Daher A (2020). Follow up of patients with severe coronavirus disease 2019 (COVID-19): Pulmonary and extrapulmonary disease sequelae. Respir. Med..

[15] Baricich A (2021). Midterm functional sequelae and implications in rehabilitation after COVID19: A cross-sectional study. Eur. J. Phys. Rehabil. Med..

[16] Tomasoni D (2021). Anxiety and depression symptoms after virological clearance of COVID-19: A cross-sectional study in Milan, Italy. J. Med. Virol..

[17] Lombardo MDM (2021). Long-term coronavirus disease 2019 complications in inpatients and outpatients: A one-year follow-up cohort study. Open Forum Infect. Dis..

[18] Xiong Q (2021). Clinical sequelae of COVID-19 survivors in Wuhan, China: A single-centre longitudinal study. Clin. Microbiol. Infect..

[19] Sonnweber T (2020). Cardiopulmonary recovery after COVID-19: An observational prospective multi-center trial. Eur. Respir. J..

[20] Gloster AT (2020). Impact of COVID-19 pandemic on mental health: An international study. PLoS ONE.

[21] Chang MC, Park D (2020). Incidence of post-traumatic stress disorder after coronavirus disease. Healthcare.

[22] Beck K (2021). Prevalence and factors associated with psychological burden in COVID-19 patients and their relatives: A prospective observational cohort study. PLoS ONE.

[23] Zhang L, Pan R, Cai Y, Pan J (2021). The prevalence of post-traumatic stress disorder in the general population during the COVID-19 pandemic: A systematic review and single-arm meta-analysis. Psychiatry Investig..

[24] Guler SA (2021). Pulmonary function and radiological features 4 months after COVID-19: First results from the national prospective observational Swiss COVID-19 lung study. Eur. Respir. J..

[25] Qin W (2021). Diffusion capacity abnormalities for carbon monoxide in patients with COVID-19 at three-month follow-up. Eur. Respir. J..

[26] Sudre CH (2021). Attributes and predictors of long COVID. Nat. Med..

[27] Ortona E, Buonsenso D, Carfi A, Malorni W (2021). Long Covid Kids study group: Long COVID: An estrogen-associated autoimmune disease?. Cell Death Discov..

[28] Bienvenu LA, Noonan J, Wang X, Peter K (2020). Higher mortality of COVID-19 in males: Sex differences in immune response and cardiovascular comorbidities. Cardiovasc. Res..

[29] Xia F, Zhang M, Cui B, Peter K (2021). COVID-19 patients with hypertension are at potential risk of worsened organ injury. Sci. Rep..

[30] Beigel JH (2020). Remdesivir for the treatment of covid-19: final report. N. Engl. J. Med..

[31] Linn BS, Linn MW, Gurel L (1968). Cumulative illness rating scale. J. Am. Geriatr. Soc..

[32] Mahler DA, Wells CK (1988). Evaluation of clinical methods for rating dyspnea. Chest.

[33] Jackson C (2015). The Chalder fatigue scale (CFQ 11). Occup. Med..

[34] Bernabeu-Mora R (2017). Determinants of each domain of the short physical performance battery in COPD. Int. J. Chron. Obstruct. Pulmon. Dis..

[35] Bernabeu-Mora R (2015). The short physical performance battery is a discriminative tool for identifying patients with COPD at risk of disability. Int. J. Chron. Obstruct. Pulmon. Dis..

[36] Leung AS, Chan KK, Sykes K, Chan KS (2006). Reliability, validity, and responsiveness of a 2-min walk test to assess exercise capacity of COPD patients. Chest.

[37] Bohannon RW, Wang YC, Gershon RC (2015). Two-minute walk test performance by adults 18 to 85 years: Normative values, reliability, and responsiveness. Arch. Phys. Med. Rehabil..

[38] Bohannon RW (2017). Normative reference values for the two-minute walk test derived by meta-analysis. J. Phys. Ther. Sci..

[39] Horowitz M, Wilner N, Alvarez W (1979). Impact of Event Scale: A measure of subjective stress. Psychosom. Med..

[40] Pietrantonio F, De Gennaro L, Paolo M, Solano L (2003). The Impact of Event Scale: Validation of an Italian version. J. Psychos. Res..

[41] Han X (2021). Six-month follow-up chest CT findings after severe covid-19 pneumonia. Radiology.

[42] Hansell DM (2008). Fleischner Society: Glossary of terms for thoracic imaging. Radiology.

