# Supplementary material for: Long-term sequelae are highly prevalent one year after hospitalization for severe COVID-19
Source: Sci Rep. 2021 Nov 22;11:22666. doi: 10.1038/s41598-021-01215-4 (PMC8608998; doi:10.1038/s41598-021-01215-4)
Supplement: Supplementary file 1 — Supplementary Information. [file 41598_2021_1215_MOESM1_ESM.docx]

**eTable 1. Comparison of pulmonary function tests at 4 and 12 months visit.** Categorical variables are shown as absolute number (%), while continuous variables are shown as medians and interquartile range [IQR]. The medians were compared by Wilcoxon’s test for paired samples, while the prevalence of DLCO reduction below the thresholds considered was tested by McNemar’s test. Abbreviations: PFTs: pulmonary function tests; FEV1: forced expiratory volume in one second; FVC: forced vital capacity; TLC: total lung capacity; DLCO: diffusion capacity of the lung for carbon monoxide. N/A: not available.

| **PFTs parameter** | **4-months visit** | **12-months visit** | **p** |
| --- | --- | --- | --- |
| **FEV1, % of predicted value** | 101 [92-113] | 101 [92-112] | 0·21 |
| **FVC, % of predicted value** | 98 [90-109] | 98 [90-109] | 0·18 |
| **TLC, % of predicted value** | N/A | 102 [94-107] | - |
| **DLCO, % of predicted value** | 79 [70-89] | 80 [69-91] | 0·17 |
| **DLCO ≥ 80%**  **DLCO < 80%** | 92 (48·7)  95 (51·3) | 100 (51·0)  96 (49·0) | 0·64 |
| **DLCO ≥ 60%**  **DLCO < 60%** | 166 (87·8)  23 (12·2) | 176 (89·8)  20 (10·2) | 0·42 |

**eTable 2. Comparison of different DLCO categories between 4 and 12 months visits.** We compared the result of DLCO measured at 4-months follow-up visit with the one measured after one year from hospital discharge. The table reports the result, restring the analysis to those patients with a clinically relevant decay of DLCO at 12-months (DLCO<80%; N.= 96). Four patients are not included since they were not able to complete DLCO measurement at 4 months, which was, therefore, unavailable. Abbreviations: DLCO: diffusion capacity of the lung for carbon monoxide; 4-m: 4 months; 12-m: 12 months.

|  | **DLCO at 4-m visit ≥ 80%** | **DLCO at 4-m visit 79-60%** | **DLCO at 4-m visit < 60%** | **Total** |
| --- | --- | --- | --- | --- |
| **DLCO at 12-m visit 79-60%** | 18 | 47 | 8 | 73 (79·3) |
| **DLCO at 12-m visit < 60%** | 0 | 5 | 14 | 19 (20·7) |
| **Total** | 18 (19·6) | 52 (56·5) | 22 (23·9) | 92 (100·0) |

**eTable 3. Univariate analysis for DLCO<80%.** Categorical variables are shown as absolute number (%), while continuous variables are shown as medians and interquartile range [IQR]. Abbreviations: BMI: body mass index; T2DM: Type 2 diabetes mellitus; COPD: chronic obstructive pulmonary disease; OSAS: obstructive sleep apnea syndrome; CKD: Chronic kidney disease; CIRS: cumulative illness rating scale; ICU: Intensive care Unit; CT: Computed tomography; IES: Impact of event scale; CPDI: CoViD-19 Peritraumatic Distress Index; DLCO: diffusion capacity of the lung for carbon monoxide.

|  | **DLCO < 80% (n/y)** | **p** |
| --- | --- | --- |
| **Age, years** | 57 [48-66] / 66 [53-73] | 0·002 |
| **Male gender** | 72 (72·0) / 49 (51·0) | 0·003 |
| **Obesity** | 11 (11·0) / 9 (9·4) | 0·81 |
| **BMI, Kg/m^2^** | 27·0 [24·6-31·2] / 27·6 [24·6-32·3] | 0·87 |
| **T2DM** | 13 (13·0) / 18 (18·8) | 0·24 |
| **COPD** | 5 (5·0) / 7 (7·7) | 0·56 |
| **OSAS** | 2 (2·0) / 2 (2·1) | 1·00 |
| **Arterial hypertension** | 41 (41·0) / 40 (41·7) | 1·00 |
| **Coronary Artery disease** | 4 (4·0) / 13 (13·5) | 0·02 |
| **Atrial fibrillation** | 4 (4·0) / 9 (9·4) | 0·16 |
| **CKD** | 2 (2·0) / 10 (10·4) | 0·02 |
| **Smoking (active-former)** | 15 (15·0) – 28 (28·0) /  8 (8·3) – 36 (37·5) | 0·19 |
| **CIRS** | 2 [1-2] / 2 [2-3] | 0·002 |
| **ICU admission** | 5 (5·0) / 18 (18·8) | 0·003 |
| **Modality of oxygen delivery**   - **nasalcannula or Venturi mask** - **Non invasive ventilation** - **Mechanical ventilation** | 45 (45·0) / 37 (38·5)  15 (15·0) / 23 (24·0)  4 (4·0) / 14 (14·6) | 0·01 |
| **Lenght of hospital in-stay, days** | 7 [5-10] /12 [8-23] | <0·0001 |
| **Acute illness severity class**   - **3** - **4** - **5** - **6** - **7)** | 27 (27·0) / 18 (18·8)  6 (6·0) / 4 (4·2)  47 (47·0) / 31 (32·3)  16 (16·0) / 26 (27·1)  4 (4·0) / 17 (17·7) | 0·003 |
| **CT severity score**   - **Mild** - **Moderate** - **Severe** | 89 (86·5) / 61 (67·0)  13 (13·5) / 13 (14·3)  0 (0·0) / 17 (18·7) | <0·001 |
| **IES** | 5 [1-15] / 10 [2-26] | 0·02 |
| **Peristent fatigue** | 13 (13·4) / 17 (17·9) | 0.39 |
| **Persistent dyspnea** | 4 (4·0) / 12 (12·5) | 0.03 |

**eTable 4. Univariate analysis for DLCO<60%.** Categorical variables are shown as absolute number (%), while continuous variables are shown as medians and interquartile range [IQR]. Abbreviations: BMI: body mass index; T2DM: Type 2 diabetes mellitus; COPD: chronic obstructive pulmonary disease; OSAS: obstructive sleep apnea syndrome; CKD: Chronic kidney disease; CIRS: cumulative illness rating scale; ICU: Intensive care Unit; CT: Computed tomography; IES: Impact of event scale; CPDI: CoViD-19 Peritraumatic Distress Index; DLCO: diffusion capacity of the lung for carbon monoxide.

|  | **DLCO < 60% (n/y)** | **p** |
| --- | --- | --- |
| **Age, years** | 61 [51-70] / 62 [50-73] | 0·49 |
| **Male gender** | 109 (61·9) / 12 (60·0) | 1·00 |
| **Obesity** | 17 (9·7) / 3 (15·0) | 0·43 |
| **BMI, Kg/m^2^** | 27·5 [24·7-31·4] / 26·0 [23·2-33·2] | 0·79 |
| **T2DM** | 23 (13·1) / 8 (40·0) | 0·005 |
| **COPD** | 8 (4·5) / 4 (20·0) | 0·02 |
| **OSAS** | 3 (1·7) / 1 (5·0) | 0·35 |
| **Arterial hypertension** | 68 (38·6) / 13 (65·0) | 0·03 |
| **Coronary Artery disease** | 14 (8·0) / 3 (15·0) | 0·39 |
| **Atrial fibrillation** | 11 (6·2) / 2 (10·0) | 0·63 |
| **CKD** | 6 (3·4) / 6 (30·0) | 0·0003 |
| **Smoking (active-former)** | 23 (13·1) – 54 (30·7) /  0 (7·5) – 10 (50·0) | 0·09 |
| **CIRS** | 2 [1-2·5] / 2·5 [2-3·5] | 0·0009 |
| **ICU admission** | 19 (10·8) / 4 (20·0) | 0·26 |
| **Modality of oxygen delivery**   - **Nasalcannula or Venturi mask** - **Non invasive ventilation** - **Mechanical ventilation** | 76 (43·2) / 6 (30·0)  32 (18·2) / 15 (8·5)  6 (30·0) / 3 (15·0) | 0·39 |
| **Lenght of hospital in-stay, days** | 9 [5-15] / 20 [8-28] | 0·006 |
| **Acute illness severity class**   - **3** - **4** - **5** - **6** - **7** | 43 (24·4) / 2 (10·0)  7 (4·0) / 3 (15·0)  72 (40·9) / 6 (30·0)  36 (20·5) / 6 (30·0)  18 (10·2) / 3 (15·0) | 0·11 |
| **CT severity score**   - **Mild** - **Moderate** - **Severe** | 135 (79·9) / 9 (50·0)  22 (13·0) / 4 (22·2)  12 (7·1) / 5 (27·8) | 0·001 |
| **IES** | 7·0 [1·5-20·0] / 4·0 [1·0-26·0] | 0·70 |

**eTable 5. Univariate analysis for functional impairment.** Categorical variables are shown as absolute number (%), while continuous variables are shown as medians and interquartile range [IQR]. Abbreviations: BMI: body mass index; T2DM: Type 2 diabetes mellitus; COPD: chronic obstructive pulmonary disease; OSAS: obstructive sleep apnea syndrome; CKD: Chronic kidney disease; CIRS: cumulative illness rating scale; ICU: Intensive care Unit; CT: Computed tomography; IES: Impact of event scale; CPDI: CoViD-19 Peritraumatic Distress Index; DLCO: diffusion capacity of the lung for carbon monoxide.

|  | **Motor impairment (n/y)** | **p** |
| --- | --- | --- |
| **Age, years** | 60 [50-69] / 69 [56-77] | 0·0004 |
| **Male gender** | 101 (68·7) / 21 (41·2) | 0·0008 |
| **Obesity** | 13 (8·8) / 8 (15·7) | 0·19 |
| **BMI, Kg/m^2^** | 27·4 [24·6-31·0] / 28·0 [25·0-34·4] | 0·06 |
| **T2DM** | 18 (12·2) / 13 (25·5) | 0·04 |
| **COPD** | 5 (3·4) / 7 (13·7) | 0·01 |
| **OSAS** | 2 (1·4) / 3 (5·9) | 0·11 |
| **Arterial hypertension** | 52 (35·4) / 30 (58·8) | 0·005 |
| **Coronary Artery disease** | 12 (8·2) / 6 (11·8) | 0·41 |
| **Atrial fibrillation** | 7 (4·8) / 6 (11·8) | 0·10 |
| **CKD** | 7 (4·8) / 5 (9·8) | 0·19 |
| **Smoking (active-former)** | 21 (14·3) – 49 (33·3) /  2 (3·9) – 17 (33·3) | 0·12 |
| **CIRS** | 2 [1-2] / 2·5 [2-3] | 0·0004 |
| **ICU admission** | 18 (12·2) / 5 (9·8) | 0·80 |
| **Modality of oxygen delivery**   - **Nasalcannula or Venturi mask** - **Non invasive ventilation** - **Mechanical ventilation** | 61 (41·5) / 20 (39·2)  31 (21·1) / 9 (17·6)  15 (10·2)/ 3 (5·9) | 0·51 |
| **Lenght of hospital in-stay, days** | 9 [5-15] / 10 [6-18] | 0·66 |
| **Acute illness severity class**   - **3** - **4** - **5** - **6** - **7** | 32 (21·8) / 14 (27·5)  6 (4·1) / 4 (7·8)  57 (38·8) / 20 (39·2)  35 (23·8) / 9 (17·6)  17 (11·6) / 4 (7·8) | 0·60 |
| **CT severity score**   - **Mild** - **Moderate** - **Severe** | 111 (79·3) / 33 (68·7)  16 (11·4) / 10 (20·8)  13 (9·3) / 5 (10·4) | 0·28 |
| **IES** | 6·0 [1·0-18·0] / 8·0 [1·5-26·0] | 0·14 |
| **DLCO** | 85 [75-92] / 69 [56-77] | <0·0001 |
| **Arthralgia/ myalgia** | 24 (16·6) / 19 (37·3) | 0.002 |

**eTable 6. Univariate analysis for moderate to severe PTS symptoms according to the IES.** Categorical variables are shown as absolute number (%), while continuous variables are shown as medians and interquartile range [IQR]. Abbreviations: BMI: body mass index; T2DM: Type 2 diabetes mellitus; COPD: chronic obstructive pulmonary disease; OSAS: obstructive sleep apnea syndrome; CKD: Chronic kidney disease; CIRS: cumulative illness rating scale; ICU: Intensive care Unit; CT: Computed tomography; DLCO: diffusion capacity of the lung for carbon monoxide.

|  | **IES > 25 (n/y)** | **p** |
| --- | --- | --- |
| **Age, years** | 62 [51-70] / 61 [52-71] | 0·97 |
| **Male gender** | 102 (64·6) / 17 (45·9) | 0·04 |
| **Obesity** | 16 (10·1) / 5 (13·5) | 0·59 |
| **BMI, Kg/m^2^** | 27·0 [24·6-31·0] / 30·4 [24·6-33·8] | 0·13 |
| **T2DM** | 28 (17·7) / 3 (8·1) | 0·21 |
| **COPD** | 9 (5·7) / 3 (8·1) | 0·70 |
| **OSAS** | 4 (2·5) / 1 (2·7) | 1·00 |
| **Arterial hypertension** | 65 (41·1) / 17 (45·9) | 0·72 |
| **Coronary Artery disease** | 15 (9·5) / 2 (5·4) | 0·75 |
| **Atrial fibrillation** | 8 (5·1) / 5 (13·5) | 0·07 |
| **CKD** | 8 (5·1) / 4 (10·8) | 0·25 |
| **Smoking (active-former)** | 20 (12·7) – 50 (31·6) /  3 (8·1) – 14 (37·8) | 0·64 |
| **CIRS** | 2 [1-3] / 2 [2-3] | 0·70 |
| **ICU admission** | 16 (10·1) / 6 (16·2) | 0·38 |
| **Modality of oxygen delivery**   - **Nasalcannula or Venturi mask** - **Non invasive ventilation** - **Mechanical ventilation** | 69 (43·7) / 11 (29·7)  32 (20·3) / 8 (21·6)  13 (8·2) / 4 (10·8) | 0·44 |
| **Lenght of hospital in-stay, days** | 9 [5-15] / 12 [7-23] | 0·05 |
| **Acute illness severity class**   - **3** - **4** - **5** - **6** - **7** | 37 (23·4) / 8 (21·6)  9 (5·7) / 0 (0·0)  63 (39·9) / 14 (37·8)  35 (22·2) / 9 (24·3)  14 (8·9) / 6 (16·2) | 0·43 |
| **CT severity score**   - **Mild** - **Moderate** - **Severe** | 116 (77·3) / 25 (71·4)  23 (15·3) / 3 (8·6)  11 (7·3) / 7 (20·0) | 0·13 |
| **DLCO** | 81 [70-92] / 72 [64-81] | 0·01 |

**eFigure 1. Representative panel of CT score interpretation.** Quantitative CT score for the evaluation of lobar fibrotic changes in 1-year follow-up of COVID19-related pneumonia. In (a) there are no fibrotic changes (0); in (b) there are about 1-10% (1) of fibrotic changes in the RUL; in (c) about 11-25% (2) of fibrotic changes in the RLL; in (d) about 26-50% (3) of fibrotic changes in the LUL and in the RUL; in (e) about 51-75% (4) of fibrotic changes in the LLL; in (f) about 76-100% (5) of fibrotic changes in the RLL.

RUL = right upper lobe; RLL = right lower lobe; LUL = left upper lobe; LLL = left lower lobe.

**
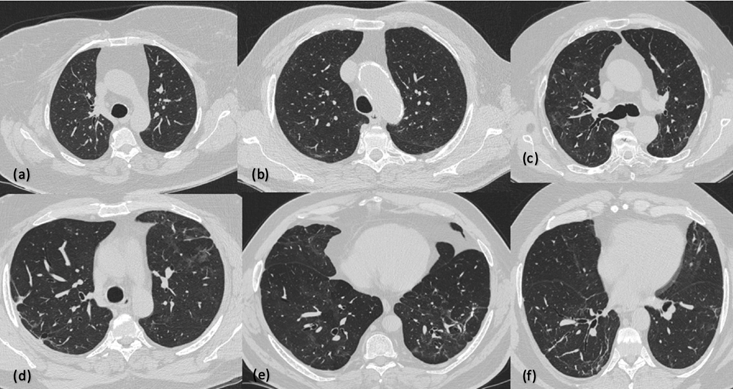
**

**eFigure 2. Variation of DLCO from first and second determination.** We compared the result of DLCO measured at 4-months follow-up visit with the one measured after one year from hospital discharge. We considered stable a variation within the range of +/- 5% of predicted value between the two determinations. We considered clinically relevant a variation > 5%. The table reports the result, restring the analysis to those patients with a clinically relevant decay of DLCO at 12-months (DLCO<80%; N.= 96). Four patients are not included since they were not able to complete DLCO measurement at 4 months, which was, therefore, unavailable. Abbreviations: DLCO: diffusion capacity of the lung for carbon monoxide.

**
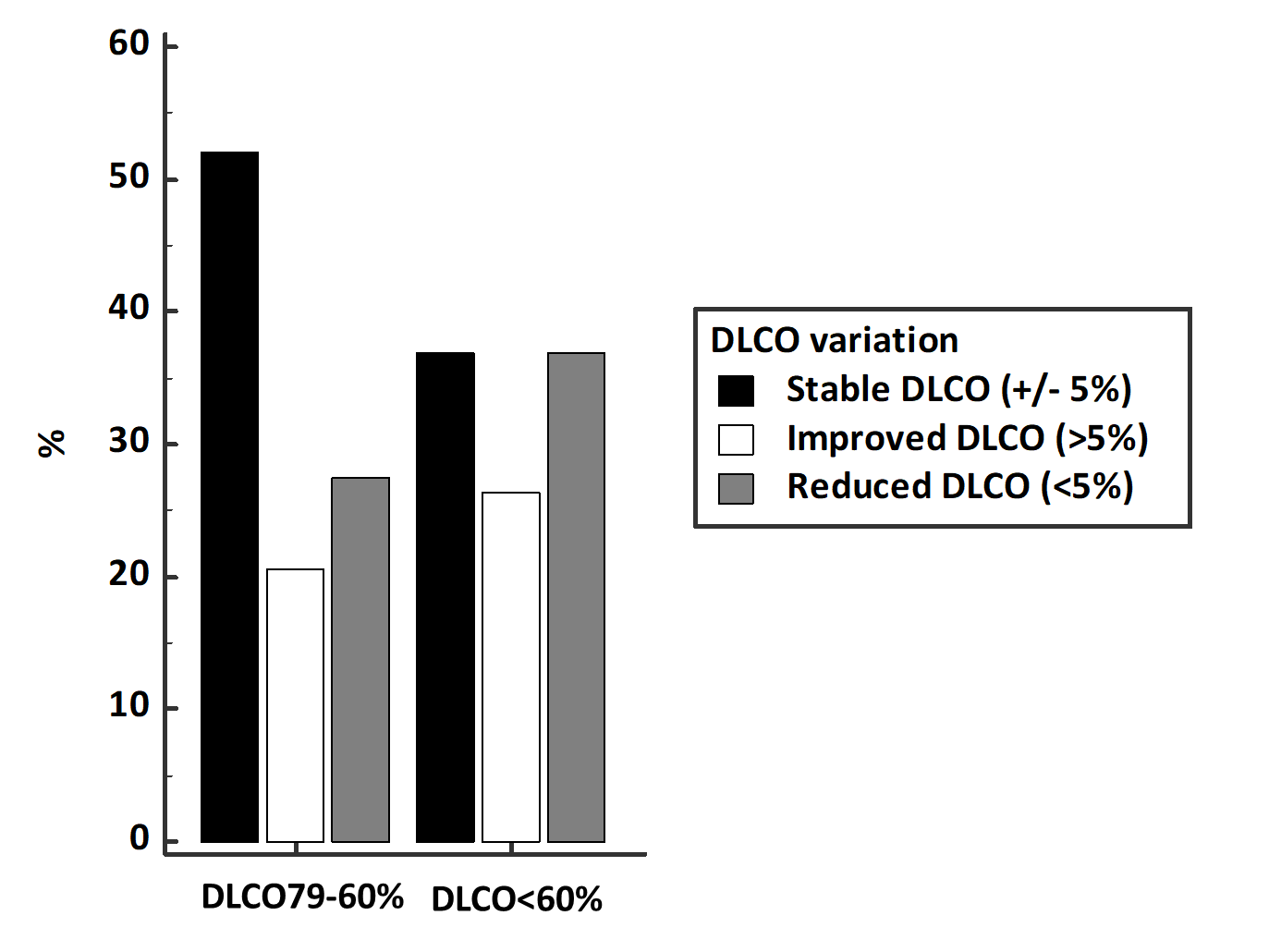
**
